# The Value of Preseason Screening for Injury Prediction: The Development and Internal Validation of a Multivariable Prognostic Model to Predict Indirect Muscle Injury Risk in Elite Football (Soccer) Players

**DOI:** 10.1186/s40798-020-00249-8

**Published:** 2020-05-27

**Authors:** Tom Hughes, Richard D. Riley, Michael J. Callaghan, Jamie C. Sergeant

**Affiliations:** 1 Manchester United Football Club, AON Training Complex, Birch Road, Off Isherwood Road, Carrington, Manchester, M31 4BH UK; 2 Centre for Epidemiology Versus Arthritis, Centre for Musculoskeletal Research, Manchester Academic Health Science Centre, University of Manchester, Manchester, UK; 3 Centre for Prognosis Research, School of Primary, Community and Social Care, Keele University, Staffordshire, UK; 4 Department of Health Professions, Manchester Metropolitan University, Brooks Building, Bonsall Street, Manchester, UK; 5 Centre for Biostatistics, University of Manchester, Manchester Academic Health Science Centre, Manchester, UK

**Keywords:** Athlete, Athletic injury, Injury prevention, Risk, Sport, Sprains and strains

## Abstract

**Background:**

In elite football (soccer), periodic health examination (PHE) could provide prognostic factors to predict injury risk.

**Objective:**

To develop and internally validate a prognostic model to predict individualised indirect (non-contact) muscle injury (IMI) risk during a season in elite footballers, only using PHE-derived candidate prognostic factors.

**Methods:**

Routinely collected preseason PHE and injury data were used from 152 players over 5 seasons (1st July 2013 to 19th May 2018). Ten candidate prognostic factors (12 parameters) were included in model development. Multiple imputation was used to handle missing values. The outcome was any time-loss, index indirect muscle injury (I-IMI) affecting the lower extremity. A full logistic regression model was fitted, and a parsimonious model developed using backward-selection to remove factors that exceeded a threshold that was equivalent to Akaike’s Information Criterion (alpha 0.157). Predictive performance was assessed through calibration, discrimination and decision-curve analysis, averaged across all imputed datasets. The model was internally validated using bootstrapping and adjusted for overfitting.

**Results:**

During 317 participant-seasons, 138 I-IMIs were recorded. The parsimonious model included only age and frequency of previous IMIs; apparent calibration was perfect, but discrimination was modest (C-index = 0.641, 95% confidence interval (CI) = 0.580 to 0.703), with clinical utility evident between risk thresholds of 37–71%. After validation and overfitting adjustment, performance deteriorated (C-index = 0.589 (95% CI = 0.528 to 0.651); calibration-in-the-large = − 0.009 (95% CI = − 0.239 to 0.239); calibration slope = 0.718 (95% CI = 0.275 to 1.161)).

**Conclusion:**

The selected PHE data were insufficient prognostic factors from which to develop a useful model for predicting IMI risk in elite footballers. Further research should prioritise identifying novel prognostic factors to improve future risk prediction models in this field.

**Trial registration:**

NCT03782389

## Key Points

- Factors measured through preseason screening generally have weak prognostic strength for future indirect muscle injuries, and further research is needed to identify novel, robust prognostic factors.
- Because of sample size restrictions and until the evidence base improves, it is likely that any further attempts at creating a prognostic model at individual club level would also suffer from poor performance.
- The value of using preseason screening data to make injury predictions or to select bespoke injury prevention strategies remains to be demonstrated, so screening should only be considered as useful for detection of salient pathology or for rehabilitation/performance monitoring purposes at this time.

## Background

In elite football (soccer), indirect (non-contact) muscle injuries (IMIs) predominantly affect the lower extremities and account for 30.3 to 47.9% of all injuries that result in time lost to training or competition [1–5]. Reduced player availability negatively impacts upon medical [6] and financial resources [7, 8] and has implications for team performance [9]. Therefore, injury prevention strategies are important to professional teams [9].

Periodic health examination (PHE), or screening, is a key component of injury prevention practice in elite sport [10]. Specifically, in elite football, PHE is used by 94% of teams and consists of medical, musculoskeletal, functional and performance tests that are typically evaluated during preseason and in-season periods [11]. PHE has a rehabilitation and performance monitoring function [12] and is also used to detect musculoskeletal or medical conditions that may be dangerous or performance limiting [13]. Another perceived role of PHE is to recognise and manage factors that may increase, or predict, an athlete’s future injury risk [10], although this function is currently unsubstantiated [13].

PHE-derived variables associated with particular injury outcomes (such as IMIs) are called prognostic factors [14], which can be used to identify risk differences between players within a team [12]. Single prognostic factors are unlikely to satisfactorily predict an individual’s injury risk if used independently [15]. However, several factors could be combined in a multivariable prognostic prediction model to offer more accurate personalised risk estimates for the occurrence of a future event or injury [15, 16]. Such models could be used to identify high-risk individuals who may require an intervention that is designed to reduce risk [17], thus assisting decisions in clinical practice [18]. Despite the potential benefits of using prognostic models for injury risk prediction, we are unaware of any that have been developed using PHE data in elite football [19].

Therefore, the aim of this study was to develop and internally validate a prognostic model to predict individualised IMI risk during a season in elite footballers, using a set of candidate prognostic factors derived from preseason PHE data.

## Methods

The methods have been described in a published protocol [20] so will only be briefly outlined. This study has been registered on ClinicalTrials.gov (identifier: NCT03782389) and is reported according to the Transparent Reporting of a Multivariable Prediction Model for Individual Prognosis or Diagnosis (TRIPOD) statement [21, 22].

### Data Sources

This study was a retrospective cohort design. Eligible participants were identified from a population of male elite footballers, aged 16–40 years old at Manchester United Football Club. A dataset was created using routinely collected injury and preseason PHE data over 5 seasons (1st July 2013 to 19th May 2018). For each season, which started on 1st July, participants completed a mandatory PHE during week 1 and were followed up to the final first team game of the season. If eligible participants were injured at the time of PHE, a risk assessment was completed by medical staff. Only tests that were appropriate and safe for the participant’s condition were completed; examiners were not blinded to injury status.

### Participants and Eligibility Criteria

During any season, participants were eligible if they (1) were not a goalkeeper and (2) participated in PHE for the relevant season. Participants were excluded if they were not contracted to the club for the forthcoming season at the time of PHE.

### Ethics and Data Use

Informed consent was not required as data were captured from the mandatory PHE completed through the participants’ employment. The data usage was approved by the Club and University of Manchester Research Ethics Service.

### Outcome

The outcome was any time-loss, index IMI (I-IMI) of the lower extremity. That is, any I-IMI sustained by a participant during matches or training, which affected lower abdominal, hip, thigh, calf or foot muscle groups and prohibited future football participation [23]. I-IMIs were graded by a club doctor or physiotherapist according to the validated Munich Consensus Statement for the Classification of Muscle Injuries in Sport [24, 25], during routine assessments undertaken within 24 h of injury. These healthcare professionals were not blinded to PHE data.

### Sample Size

We allowed a maximum of one candidate prognostic factor parameter per 10 I-IMIs, which at the time of protocol development, was the main recommendation to minimise overfitting (Additional file 1) [20, 26]. The whole dataset was used for model development and internal validation, which agrees with methodological recommendations [27].

### Candidate Prognostic Factors

The available dataset contained 60 candidate factors [20]. Because of the sample size considerations, before any analysis, the set of candidate factors was reduced. Initially, an audit was conducted to quantify missing values and to determine the measurement reliability of the eligible candidate factors [20]. Any candidate factors which had greater than 15% missing data or where reliability was classed as fair to poor (intraclass correlation coefficient < 0.70) were excluded [20] (Additional file 2). Of the remaining 45 eligible factors, previous evidence of prognostic value [19] and clinical reasoning were used to select candidate prognostic factors suitable for inclusion [20]. This process left a final set of 10 candidate factors, represented by 12 model parameters (Table 1). The 35 factors that were not included in model development are also listed in Additional File 2, and will be utilised in a related, forthcoming exploratory study which aims to examine their association with indirect muscle injuries in elite football players.

**Table 1:** Set of candidate prognostic factors (with corresponding number of parameters) for model development

Selection method	Candidate prognostic factor	Measurement unit	Number of model parameters corresponding to PF	Measurement method	Data type	Reliability (if applicable)
Systematic review/clinical reasoning	Age	Years and days	1	Date of birth	Continuous	N/A
	Frequency of previous IMIs within 3 years prior to PHE	Count	1	Medical records	Discrete (treated as continuous)	N/A
	Most recent previous IMI within 3 years prior to baseline PHE	Never (ref); < 6 months; 6–12 months; > 12 months	3	Medical records	Categorical	N/A
Data quality/clinical reasoning	CMJ peak power	Watts	1	CMJ using force plates	Continuous	Test-retest ICC = 0.92–0.98 [28]
	PROM hip joint internal rotation difference*	Degrees	1	Supine ROM test using digital inclinometer	Continuous	Intra-rater ICC = 0.90 [29]
	PROM hip joint external rotation difference*	Degrees	1	Supine ROM test using digital inclinometer	Continuous	Intra-rater ICC = 0.90 [29]
	Hip flexor muscle length difference*	Degrees	1	Thomas test using digital inclinometer	Continuous	Inter-rater ICC = 0.89 [30]
	Hamstring muscle length/neural mobility difference*	Degrees	1	SLR using digital inclinometer	Continuous	Intra-rater ICC = 0.95–0.98 [31]; inter-rater ICC = 0.80–0.97 [32]
	Calf muscle length difference*	Degrees	1	WBL using digital inclinometer	Continuous	Inter-rater ICC = 0.80–0.95 [33, 34]; intra-rater ICC = 0.88 [34]
	BMI	kg/m^2^	1	Composite height (cm) and weight (kg)	Continuous	N/A

*PF* prognostic factor, *PHE* periodic health examination, *IMI* indirect muscle injury, *ref* reference category (does not count as a model parameter), *WBL* weight bearing lunge, *CMJ* countermovement jump, *ROM* range of movement, *PROM* passive range of movement, *ICC* intraclass correlation coefficient, *SLR* straight leg raise, *BMI* body mass index, *kg* kilos, *m* mass, *N/A* not applicable

*Denotes between limb differences

### Statistical Analysis

#### Data Handling—Outcome Measures

Each participant-season was treated as independent. Participants who sustained an I-IMI were no longer considered at risk for that season and were included for further analysis at the start of the next season if still eligible. Any upper limb IMI, trunk IMI or non-IMI injuries were ignored, and participants were still considered at risk.

Eligible participants who were loaned to another club throughout that season, but had not sustained an I-IMI prior to the loan, were still considered at risk. I-IMIs that occurred whilst on loan were included for analysis, as above. Permanently transferred participants (who had not sustained an I-IMI prior to leaving) were recorded as not having an I-IMI during the relevant season and exited the cohort at the season end.

#### Data Handling—Missing Data

Missing values were assumed to be missing at random [20]. The continuous parameters generally demonstrated non-normal distributions, so were transformed using normal scores [35] to approximate normality before imputation, and back-transformed following imputation [36]. Multivariate normal multiple imputation was performed, using a model that included all candidates and I-IMI outcomes. Fifty imputed datasets were created in Stata 15.1 (StataCorp LLC, Texas, USA) and analysed using the *mim* module.

#### Prognostic Model Development

Continuous parameters were retained on their original scales, and their effects assumed linear [22]. A full multivariable logistic regression model was constructed, which contained all 12 parameters. Parameter estimates were combined across imputed datasets using Rubin’s Rules [37]. To develop a parsimonious model that would be easier to utilise in practice, backward variable selection was performed using estimates pooled across the imputed datasets at each stage of the selection procedure to successively remove non-significant factors with *p* values > 0.157. This threshold was selected to approximate equivalence with Akaike’s Information Criterion [38, 39]. Multiple parameters representing the same candidate factor were tested together so that the whole factor was either retained or removed. Candidate interactions were not examined, and no terms were forced into the model. All analyses were conducted in Stata 15.1.

#### Assessment of Model Performance

The full and parsimonious models were used to predict I-IMI risk over a season, for every participant-season in all imputed datasets. For all performance measures, each model’s apparent performance was assessed in each imputed dataset and then averaged across all imputed datasets using Rubin’s Rules [37]. Discrimination determines a model’s ability to differentiate between participants who have experienced an outcome compared to those who have not [40], quantified using the concordance index (C-index). This is equivalent to the area under the receiver operating characteristic (ROC) curve for logistic regression, where 1 demonstrates perfect discrimination, whilst 0.5 indicates that discrimination is no better than chance [41].

Calibration determines the agreement between the model’s predicted outcome risks and those observed [42], evaluated using an apparent calibration plot in each imputed dataset. All predicted risks were divided into ten groups defined by tenths of predicted risk. The mean predicted risks for the groups were plotted against the observed group outcome proportions with corresponding 95% confidence intervals (CIs). A loess smoothing algorithm showed calibration across the range of predicted values [43]. For grouped and smoothed data points, perfect predictions lie on the 45° line (i.e. a slope of 1).

The systematic (mean) error in model predictions was quantified using calibration-in-the-large (CITL), which has an ideal value of 0 [40, 42], and the expected/observed (E/O) statistic, which is the ratio of the mean predicted risk against the mean observed risk (ideal value of 1) [40, 42]. The degree of over or underfitting was determined using the calibration slope, where a value of 1 equals perfect calibration on average across the entire range of predicted risks [22]. Nagelkerke’s pseudo-*R*^2^ was also calculated, which quantifies the overall model fit, with a range of 0 (no variation explained) to 1 (all variation explained) [44].

#### Assessment of Clinical Utility

Decision-curve analysis was used to assess the parsimonious model’s apparent clinical usefulness in terms of net benefit (NB) if used to allocate possible preventative interventions. This assumed that the model’s predicted risks were classed as positive (i.e. may require a preventative intervention) if greater than a chosen risk threshold, and negative otherwise. NB is then the difference between the proportion of true positives and false positives, where both were weighted by the odds of the chosen risk threshold and also divided by the sample size [45]. Positive NB values suggest the model is beneficial compared to treating none, which has no benefit to the team but with no negative cost and efficiency implications. The maximum possible NB value is the proportion with the outcome in the dataset.

The model’s NB was also compared to the NB of delivering an intervention to all individuals. This is considered a treat-all strategy, offering maximum benefit to the team, but with maximum negative cost and efficiency implications [17]. A model has potential clinical value if it demonstrates higher NB than the default strategies over the range of risk thresholds which could be considered as high risk in practice [46].

#### Internal Validation and Adjustment for Overfitting

To examine overfitting, the parsimonious model was internally validated using 200 bootstrap samples, drawn from the original dataset with replacement. In each sample, the complete model-building procedure (including multiple imputation, backward variable selection and performance assessment) was conducted as described earlier. The difference in apparent performance (of a bootstrap model in its bootstrap sample) and test performance (of the bootstrap model in the original dataset) was averaged across all samples. This generated optimism estimates for the calibration slope, CITL and C-index statistics. These were subtracted from the original apparent calibration slope, CITL and C-index statistics to obtain final optimism-adjusted performance estimates. The Nagelkerke *R*^2^ was adjusted using a relative reduction equivalent to the relative reduction in the calibration slope.

To produce a final model adjusted for overfitting, the regression coefficients produced in the parsimonious model were multiplied by the optimism-adjusted calibration slope (also termed a uniform shrinkage factor), to adjust (or shrink) for overfitting [47]. Finally, the CITL (also termed model intercept) was then re-estimated to give the final model, suitable for evaluation in other populations or datasets.

#### Complete Case and Sensitivity Analyses

To determine the effect of multiple imputation and player transfer assumptions on model stability, the model development process was repeated: (1) as a complete case analysis and (2) as sensitivity analyses which excluded all participant-seasons where participants had not experienced an I-IMI up to the point of loan or transfer, which were performed as both multiple imputation and complete case analyses.

## Results

### Participants

**Fig. 1:**
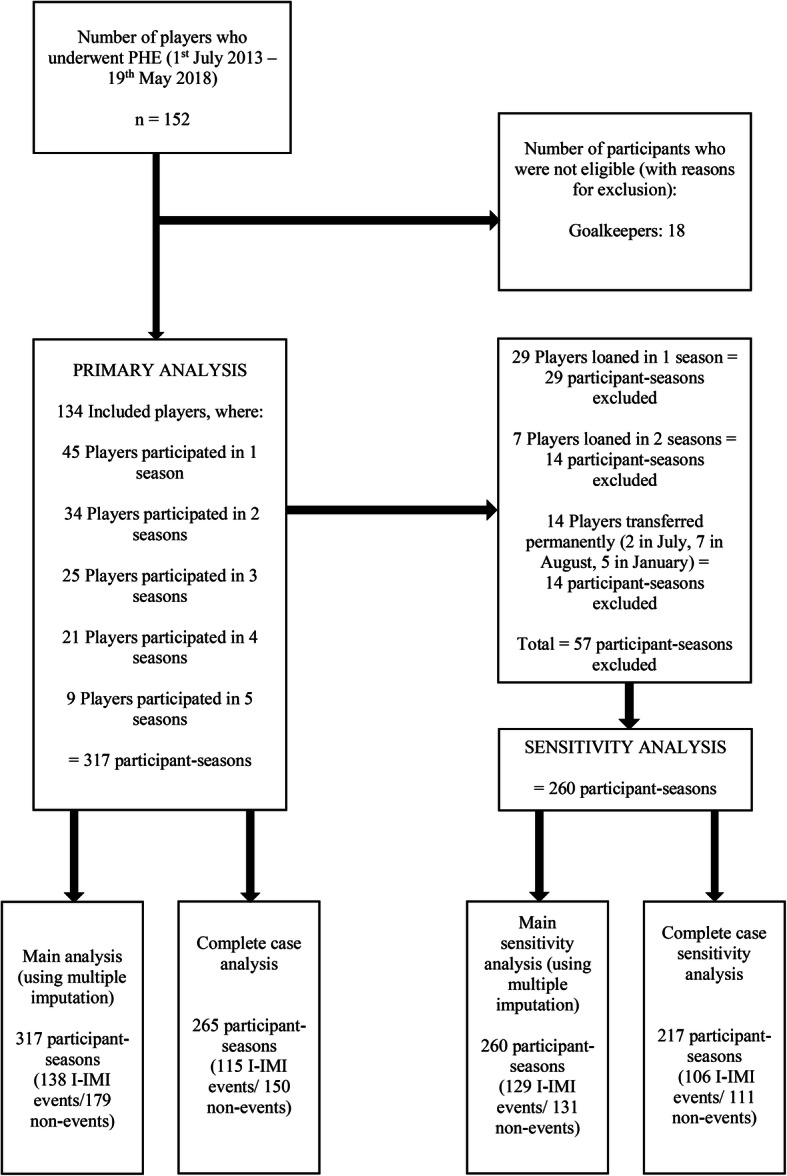
Participant flow chart. Key: *n* = participants; I-IMI = index indirect muscle injury

During the five seasons, 134 participants were included, contributing 317 participant-seasons and 138 IMIs in the primary analyses (Fig. 1). Three players were classified as injured when they took part in PHE (which affected three participant-seasons). This meant they were unavailable for full training or to play matches at that time. However, these players had commenced football specific, field-based rehabilitation around this time, so also had similar exposure to training activities as the uninjured players. As such, these players were included in the cohort because it was reasonable to assume that they could also be considered at risk of an I-IMI event even during their rehabilitation activities.

Table 2 describes the frequency of included participant-seasons, and the frequency and proportion of recorded I-IMI outcomes across all five seasons. For the sensitivity analyses (excluding loans and transfers), 260 independent participant-seasons with 129 IMIs were included; 36 participants were transferred on loan, whilst 14 participants were permanently transferred during a season, which excluded 57 participant-seasons in total (Fig. 1). Table 2 also describes the frequency of excluded participant-seasons where players were transferred either permanently or on loan, across the 5 seasons.

**Table 2:** Frequency of included participant-seasons, I-IMI outcomes and participant-seasons affected by transfers, per season (primary analysis)

			**Season**			
	**1 (2013/2014)**	**2 (2014/2015)**	**3 (2015/2016)**	**4 (2016/2017)**	**5 (2017/2018)**	**Total**
Included participant-seasons	58	66	66	61	66	317
Participant-seasons with an I-IMI outcome (%)	26 (44.83)	21 (31.82)	29 (43.93)	28 (45.90)	34 (51.52)	138 (44.00)
Participant-seasons where players transferred on loan	16	10	7	4	6	43
Participant-seasons where players transferred permanently	1	4	5	3	1	14

*I*-*IMI* index indirect muscle injury

Table 3 shows anthropometric and all prognostic factor characteristics for participants included in the primary analyses. These were similar to those included in the sensitivity analyses (Additional file 3).

**Table 3:** Characteristics of included participants in the primary analysis

Characteristic/candidate prognostic factor	Measurement method	Data type	Freq. (%) if categorical	Min	Lower quartile	Median	Upper quartile	Max	Missing values *n* (%)
**Anthropometrics**									
Age at PHE (years)	Birthdate	Cont.	-	16.01	17.80	19.69	23.56	39.59	0 (0)
Height (cm)	Standing height	Cont.	-	164.3	176.0	180.0	185.5	195.0	18 (5.68)
Weight (kg)	Digital scales	Cont.	-	56.8	69.2	73.6	80.0	94.0	18 (5.68)
BMI (kg/m^2^)	Calculated using the formula: kg/m^2^	Cont.	-	18.1	21.8	22.7	23.7	29.1	23 (7.26)
**Past medical history**									
Freq. of previous IMIs in 3 years prior to PHE	Medical records	Dis./cont.	-	0	0	1	2	7	0 (0)
Most recent previous IMI in 3 years prior to PHE									
Never	Medical records	Cat.	143 (45.11)	-	-	-	-	-	0 (0)
< 6 months	Medical records	Cat.	48 (15.14)	-	-	-	-	-	0 (0)
6–12 months	Medical records	Cat.	52 (16.40)	-	-	-	-	-	0 (0)
> 12 months	Medical records	Cat.	74 (23.34)	-	-	-	-	-	0 (0)
**Musculoskeletal examination**									
PROM hip internal rotation difference (deg.)	Supine ROM test with digital inclinometer	Cont.	-	− 25.0	− 3.0	0.0	5.0	20.0	20 (6.31)
PROM hip external rotation difference (deg.)	Supine ROM test with digital inclinometer	Cont.	-	− 20.0	− 5.0	0.0	5.0	25.0	20 (6.31)
Hip flexor length difference (deg.)	Thomas test with digital inclinometer	Cont.	-	− 20.0	− 2.0	0.0	3.0	14.0	23 (7.26)
Hamstring length/neural mobility difference (deg.)	SLR with digital inclinometer	Cont.	-	− 20.0	0.0	0.0	0.0	15.0	23 (7.26)
Calf muscle length difference (deg.)	WBL with digital inclinometer	Cont.	-	− 20.0	− 2.0	0.0	3.0	15.0	20 (6.31)
**Lower extremity power**									
CMJ power (watts)	CMJ using force platform	Cont.	-	2625.0	3707.0	4150.0	4662.0	6577.0	42 (13.25)

*PHE* periodic health examination, *I-IMI* index indirect muscle injury, *IMI* indirect muscle injury, *min* minimum, *max* maximum, *n* observations, *Freq* frequency, *WBL* weight bearing lunge, *CMJ* countermovement jump, *ROM* range of movement, *PROM* passive range of movement, *deg*. degrees, *SLR* straight leg raise, *BMI* body mass index, *kg*/*m*^2^ kilograms/body height (metres) squared, *cm* centimetres, *kg* kilograms, *Cont*. continuous, *dis*./*cont*. discrete treated as continuous, *cat*. categorical

### Missing Data and Multiple Imputation

All I-IMI, age and previous muscle injury data were complete (Table 3). For all other candidates, missing data ranged from 6.31 (for hip internal and external rotation difference) to 13.25% for countermovement jump (CMJ) power (Table 3). The distribution of imputed values approximated observed values (Additional file 4), confirming their plausibility.

### Model Development

Table 4 shows the parameter estimates for the full model and parsimonious model after variable selection (averaged across imputations).

**Table 4:** Results of the full and parsimonious multivariable logistic regression models, with prediction formulae

	Full model					Parsimonious model (after variable selection)					Final model after adjustment (shrinkage) for overfitting	
Candidate prognostic factors	β^†^	95% CI	OR	95% CI	*p* value	β^†^	95% CI	OR	95% CI	*p* value	Adjusted β^†^	Adjusted OR
**Anthropometrics**												
Age at PHE (years)	**0.095**	**0.032 to 0.159**	**1.100**	**1.032 to 1.172**	**0.003**	**0.091**	**0.034 to 0.148**	**1.095**	**1.035 to 1.159**	**0.002**	0.065*	**1.068**
BMI (kg/m^2^)	− 0.078	− 0.249 to 0.093	0.925	0.780 to 1.098	0.372							
**Past medical history**												
Freq. of previous IMIs in 3 years prior to PHE	**0.235**	− **0.037 to 0.507**	**1.265**	**0.964 to 1.661**	**0.090**	**0.168**	− **0.015 to 0.350**	**1.182**	**0.986 to 1.419**	**0.0**	0.120*	**1.128**
Most recent previous IMI in 3 years prior to PHE												
Never	Ref	Ref	Ref	Ref	Ref	-	-	-	-	-	-	-
< 6 months	0.043	− 0.892 to 0.978	1.044	0.410 to 2.660	0.928	-	-	-	-	-	-	-
6–12 months	− 0.463	− 1.317 to 0.392	0.630	0.268 to 1.480	0.289	-	-	-	-	-	-	-
> 12 months	− 0.308	− 1.056 to 0.440	0.735	0.348 to 1.553	0.420	-	-	-	-	-	-	-
**Musculoskeletal examination**											-	-
PROM hip internal rotation difference (deg.)	0.008	− 0.029 to 0.044	1.008	0.971 to 1.045	0.682	-	-	-	-	-	-	-
PROM hip external rotation difference (deg.)	0.024	− 0.011 to 0.059	1.024	0.989 to 1.061	0.180	-	-	-	-	-	-	-
Hip flexor length difference (deg.)	0.026	− 0.032 to 0.083	1.026	0.969 to 1.087	0.382	-	-	-	-	-	-	-
Hamstring length/neural mobility difference (deg.)	− 0.007	− 0.083 to 0.068	0.993	0.920 to 1.070	0.846	-	-	-	-	-	-	-
Calf muscle length difference (deg.)	0.018	− 0.033 to 0.069	1.018	0.967 to 1.072	0.493	-	-	-	-	-	-	-
**Lower extremity power**												
CMJ power (watts)	0.000	0.000 to 0.001	1.000	1.000 to 1.001	0.394	-	-	-	-	-	-	-
**Model Intercept **	− 1.448	− 4.564 to 1.668	-	-	-	− 2.384	− 3.558 to − 1.211	-	-	-	− 1.786**	-
**Model performance statistics**	**Apparent performance (95% CI)**					**Apparent performance (95% CI)—before validation**		**Optimism-adjusted performance with 95% CI—after validation**				
**Nagelkerke*R***^**2**^	0.120					0.089		0.064				
**Calibration slope**	1.000 (0.608 to 1.392)					1.000 (0.557 to 1.443)		0.718 (0.275 to 1.161)			-	-
**CITL**	0.000 (− 0.233 to 0.233)					0.000 (− 0.230 to 0.230)		− 0.009 (− 0.239 to 0.239)			-	-
**C-index**	0.670 (0.609 to 0.731)					0.641 (0.580 to 0.703)		0.589 (0.528 to 0.651)			-	-

Factors in bold indicate significance at the 0.157 level (equivalent to Akaike’s Information Criterion)

*β* Beta (regression) coefficient, *SE* standard error, *CI* confidence interval, *OR* odds ratio, *PHE* periodic health examination, *Freq.* frequency, *IMI* indirect muscle injury, *deg.* degrees, *BMI* body mass index, *kg*/*m*^2^ kilograms/body height squared, *ref* reference category

^**†**^*β* values are expressed per one-unit increase for all continuous variables, and according to category for the most recent IMI within 3 years prior to PHE

*Adjusted regression value after multiplication with uniform shrinkage factor of 0.718

**Re-estimated model intercept after internal validation

Prediction formula of parsimonious model (used during internal validation procedure). The predicted probability of a player sustaining an I-IMI during a season can be calculated using the following: Probability = 1/(1 + exp(2.384 − 0.091 × age − 0.168 × freq. of previous IMIs within 3 years prior to PHE)). If desired, a percentage risk score can be obtained by multiplying the probability × 100.

Prediction formula for final model (for use on new datasets). The predicted probability of a player sustaining an I-IMI during a season can be calculated using the following: Probability = 1/(1 + exp(1.786 − 0.065 × age − 0.120 × freq. of previous IMIs within 3 years prior to PHE)). Note: exp = exponentiate. If desired, a percentage risk score can be obtained by multiplying the probability × 100

For both models, only age and frequency of previous IMIs had a statistically significant (but modest) association with increased I-IMI risk (*p* < 0.157). No clear evidence for an association was observed for any other candidate factor.

### Model Performance Assessment and Clinical Utility

**Fig. 2:**
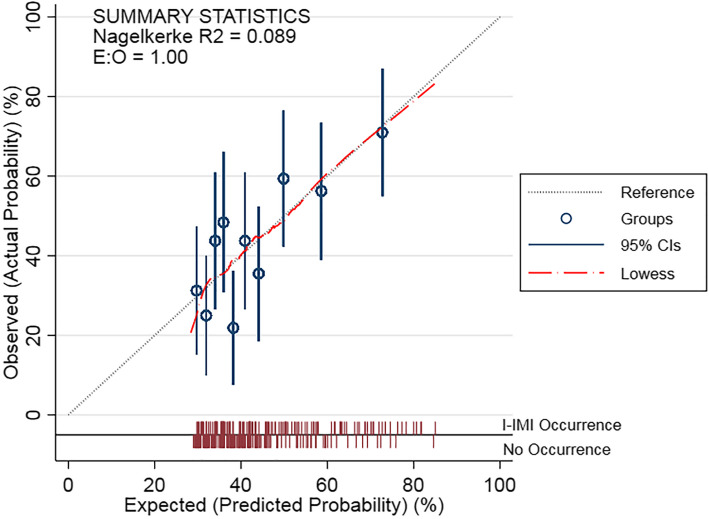
Apparent calibration of the parsimonious model (before adjustment for overfitting). Key: E:O = expected to observed ratio; CI = confidence interval; I-IMI = index indirect muscle injury

Table 4 shows the apparent performance measures for the full and parsimonious models, all of which were similar. Figure 2 shows the apparent calibration of the parsimonious model in the dataset used to develop the model (i.e. before adjustment for overfitting). These were identical across all imputed datasets because the retained prognostic factors contained no missing values. The parsimonious model had perfect apparent overall CITL and calibration slope by definition, but calibration was more variable around the 45° line between the expected risk ranges of 28 to 54%. Discrimination was similarly modest for the full (C-index = 0.670, 95% CI = 0.609 to 0.731) and parsimonious models (C-index = 0.641, 95% CI = 0.580–0.703). The apparent overall model fit was low for both models, indicated by Nagelkerke *R*^2^ values of 0.120 for the full model and 0.089 for the parsimonious model.

**Fig. 3:**
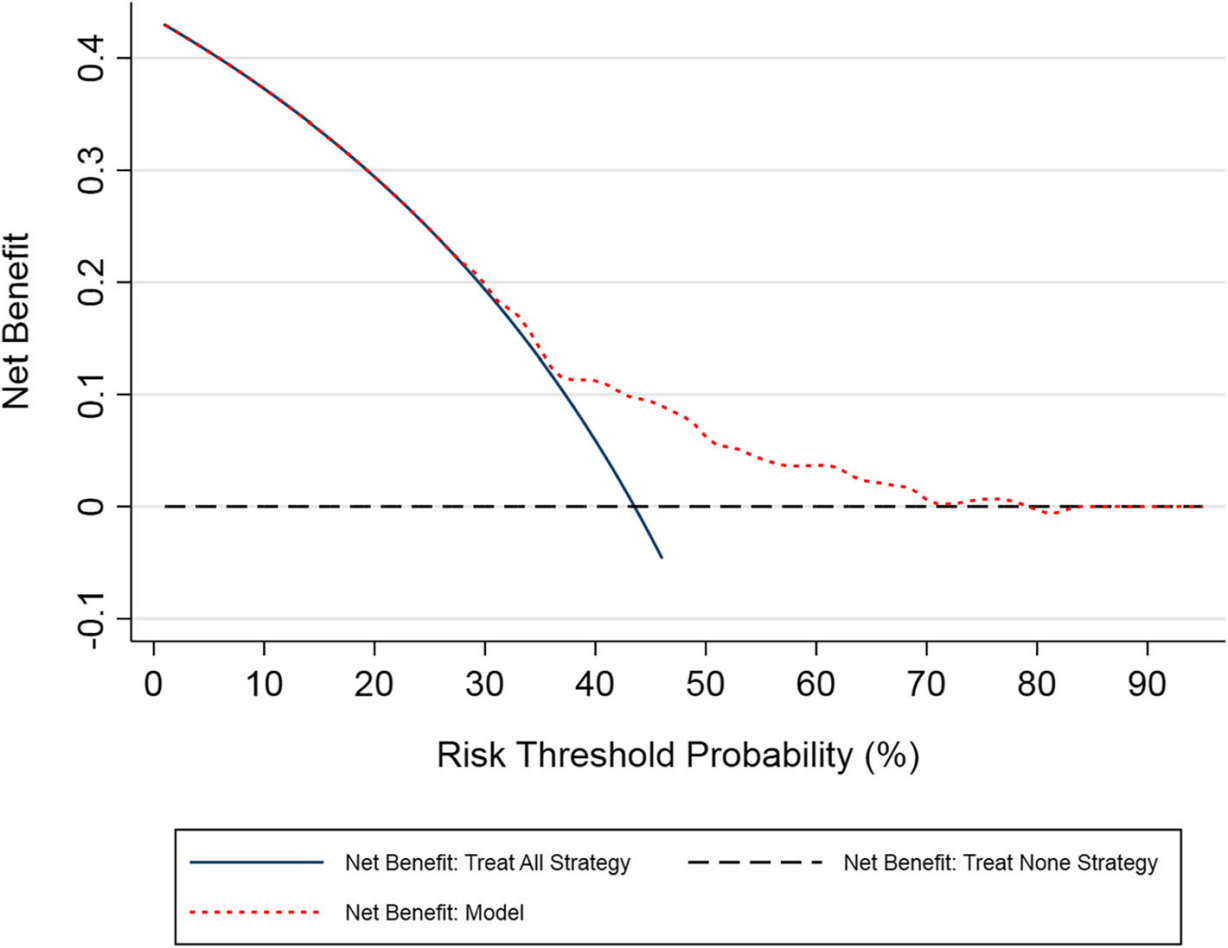
Decision curve analysis for the parsimonious model (before adjustment for overfitting)

Figure 3 displays the decision-curve analysis. The NB of the parsimonious model was comparable to the treat-all strategy at risk thresholds up to 31%, marginally greater between 32 and 36% and exceeded the NB of either default strategies between 37 and 71%.

### Internal Validation and Adjustment for Overfitting

Table 4 shows the optimism-adjusted performance statistics for the parsimonious model, with full internal validation results shown in Additional file 9. After adjustment for optimism, the overall model fit and the model’s discrimination performance deteriorated (Nagelkerke *R*^2^ = 0.064; C-index = 0.589 (95% CI = 0.528 to 0.651). Furthermore, bootstrapping suggested the model would be severely overfitted in new data (calibration slope = 0.718 (95% CI = 0.275 to 1.161)), so a shrinkage factor of 0.718 was applied to the parsimonious parameter estimates, and the model intercept re-estimated to produce our final model (Table 4).

### Complete Case and Sensitivity Analyses

The full and parsimonious models were robust to complete case analyses and excluding loans and transfers, with comparable apparent performance estimates. For the full models, the C-index range was 0.675 to 0.705, and Nagelkerke *R*^2^ range was 0.135 to 0.178, whilst for the parsimonious models, the C-index range was 0.632 to 0.691, and Nagelkerke *R*^2^ range was 0.102 to 0.154 (Additional files 5, 6, 7, 8 and 9). The same prognostic factors were selected in all parsimonious models. The degree of estimated overfitting observed in the complete case and sensitivity analyses was comparable to that observed in the main analysis (calibration slope range = 0.678 to 0.715) (Additional files 5, 6, 7, 8 and 9).

## Discussion

We have developed and internally validated a multivariable prognostic model to predict individualised I-IMI risk during a season in elite footballers, using routinely, prospectively collected preseason PHE and injury data that was available at Manchester United Football Club. This is the only study that we know of that has developed a prognostic model for this purpose, so the results cannot be compared to previous work.

We included both a full model which did not include variable selection and a parsimonious model, which included a subset of variables that were statistically significant. The full model was included because overfitting is likely to increase when variable inclusion decisions are based upon *p* values. In addition, the use of *p* value thresholds for variable selection is somewhat arbitrary. However, the overfitting that could have arisen in the parsimonious model after using *p* values in this way was accounted for during the bootstrapping process, which replicated the variable selection strategy based on *p* values in each bootstrap sample.

The performance of the full and parsimonious models was similar, which means that utilising all candidate factors offered very little advantage over using two for making predictions. Indeed, variable selection eliminated 8 candidate prognostic factors that had no clear evidence for an association with I-IMIs. Our findings confirm previous suggestions that PHE tests designed to measure modifiable physical and performance characteristics typically offer poor predictive value [10]. This may be because unless particularly strong associations are observed between a PHE test and injury outcome, the overlap in scores between individuals who sustain a future injury and those who do not results in poor discrimination [10]. Additionally, after measurement at a single timepoint (i.e. preseason), it is likely that the prognostic value of these modifiable factors may vary over time [48] due to training exposure, environmental adaptations and the occurrence of injuries [49].

The variable selection process resulted in a model which included only age and the frequency of previous IMIs within the last 3 years, which are simple to measure and routinely available in practice. Our findings were similar to the modest association previously observed between age and hamstring IMIs in elite players [19]. However, whilst a positive previous hamstring IMI history has a confirmed association with future hamstring IMIs [19], we found that for lower extremity I-IMIs, cumulative IMI frequency was preferred to the time proximity of any previous IMI as a multivariable prognostic factor. Nevertheless, the weak prognostic strength of these factors explains the parsimonious model’s poor discrimination and low potential for clinical utility.

Our study is the first to utilise decision-curve analysis to examine the clinical usefulness of a model for identifying players at high risk of IMIs and who may benefit from preventative interventions such as training load management, strength and conditioning or physiotherapy programmes. Our parsimonious model demonstrated no clinical value at risk thresholds of less than 36%, because its NB was comparable to that of providing all players with an intervention. Indeed, the only clinically useful thresholds that would indicate a high-risk player would be 37–71%, where the model’s NB was greater than giving all players an intervention. However, because of the high baseline IMI risk in our population (approximately 44% of participant-seasons affected), the burden of IMIs [1–5] and the minimal costs [10] versus the potential benefits of such preventative interventions in an elite club setting, these thresholds are likely to be too high to be acceptable in practice. Accordingly, it would be inappropriate to allocate or withhold interventions based upon our model’s predictions.

Because of severe overfitting our parsimonious model was optimistic, which means that if used with new players, prediction performance is likely to be worse [39]. Although our model was adjusted to account for overfitting and hence improve its calibration performance in new datasets, given the limitations in performance and clinical value, we cannot recommend that it is validated externally or used in clinical practice.

This study has some limitations. We acknowledge that the development of our model does not formally take account of the use of existing injury prevention strategies, including those informed by PHE, and their potential effects on the outcome. Rather, we predicted I-IMIs under typical training and match exposure and under routine medical care. In addition, it should be noted that injury risk predictions at an elite level football club may not generalise to other types of football clubs or sporting institutions, where ongoing injury prevention strategies may not be comparable in terms of application and equipment.

We measured candidate factors at one timepoint each season and assumed that participant-seasons were independent. Whilst statistically complex, future studies may improve predictive performance and external validity by harnessing longitudinal measurements and incorporating between-season correlations.

We did not perform a competing risks analysis to account for players not being exposed to training and match play due to injuries other than I-IMIs. That is, our approach predicted the risk of I-IMIs in the follow up of players, allowing other injury types to occur and therefore possibly limiting the opportunity for I-IMIs during any rehabilitation period. The competing risk of the occurrence of non-IMIs was therefore not explicitly modelled and players remained in the risk set after a non-IMI had occurred.

We also merged all lower extremity I-IMIs rather than using specific muscle group outcomes. Although less clinically meaningful, this was necessary to maximise statistical power. Nevertheless, our limited sample size prohibited examination of complex non-linear associations and only permitted a small number of candidates to be considered. A lack of known prognostic factors [19] meant that selection was mainly guided by data quality control processes and clinical reasoning, so it is possible that important factors were not included.

Risk prediction improves when multiple factors with strong prognostic value are used [15]. Therefore, future research should aim to identify novel prognostic factors, so that these can be used to develop models with greater potential clinical benefit. This may also allow updating of our model to improve its performance and clinical utility [50].

Until the evidence base improves, and because of sample size limitations, it is likely that any further attempts to create a prognostic model at individual club level would suffer similar issues. Importantly, this means that for any team, the value of using preseason PHE data to make individualised predictions or to select bespoke injury prevention strategies remains to be demonstrated. However, the pooling of individual participant data from several participating clubs may increase sample sizes sufficiently to allow further model development studies [51], where a greater number of candidate factors could be utilised.

## Conclusion

Using PHE and injury data available preseason, we have developed and internally validated a prognostic model to predict I-IMI risk in players at an elite club, using current methodological best practice. The paucity of known prognostic factors and data requirements for model building severely limited the model’s performance and clinical utility, so it cannot be recommended for external validation or use in practice. Further research should prioritise identifying novel prognostic factors to improve future risk prediction models in this field.

## Supplementary information

**Additional file 1.** Sample size calculation.

**Additional file 2.** Candidate prognostic factors that were excluded from the analysis, with reasons for exclusion.

**Additional file 3.** Anthropometric parameters and all included candidate PFs characteristics for participants included in the sensitivity analysis.

**Additional file 4.** Graph to show the log-transformed distribution of observed and imputed values for continuous variables.

**Additional file 5.** Results of the full multivariable logistic regression model and the model after variable selection – Primary complete case analysis.

**Additional file 6.** Results of the full multivariable logistic regression model and the model after variable selection – Sensitivity analysis using imputed data.

**Additional file 7.** Results of the full multivariable logistic regression model and the model after variable selection – Sensitivity analysis using complete case data.

**Additional file 8.** Apparent calibration plots for primary complete case analysis and sensitivity analyses.

**Additional file 9.** Full internal validation results for all analyses.

## Data Availability

An anonymised summary of the dataset that was analysed during this study may be available from the corresponding author on reasonable request.
