# Supplementary material for: The Value of Preseason Screening for Injury Prediction: The Development and Internal Validation of a Multivariable Prognostic Model to Predict Indirect Muscle Injury Risk in Elite Football (Soccer) Players
Source: Sports Med Open. 2020 May 27;6:22. doi: 10.1186/s40798-020-00249-8 (PMC7253524; doi:10.1186/s40798-020-00249-8)
Supplement: Supplementary file 1 — Additional file 1. Sample size calculation. [file 40798_2020_249_MOESM1_ESM.pdf]

Additional file 1

The value of pre-season screening for injury prediction: The development and internal validation of a multivariable prognostic model to predict indirect muscle injury risk in elite football (soccer) players. Sports Medicine - Open.

Hughes, T., Riley, R.D. Sergeant, J.C., Callaghan, M.J. (2020)

Corresponding author: Tom Hughes

Email: tom.hughes.physio@manutd.co.uk

Correspondence address: Manchester United Football Club, AON Training Complex, Birch Road, Off Isherwood Road, Carrington, Manchester. UK. M31 4BH.
Tel: 0161 868 8754

Sample size calculation

Prognostic models suffer from optimism, where prediction performance is worse when applied in datasets that are not considered during development [1]. This is primarily due to overfitting, where the number of included candidate PFs is too large relative to the number of outcomes, or if irrelevant candidate PFs are included [2]. Because our sample size was fixed, to determine the maximum number of candidate PFs for inclusion, we used a minimum of 10 events per variable (EPV) rule, recommended to reduce statistical overfitting of logistic regression models [3]. Note that 'variable' means any parameter included (or considered for inclusion) that corresponded to a PF.

During the 5-seasons, 138 I-IMIs were recorded. We restricted the number of included parameters to 12, which corresponded to >10 EPV. This also met the criteria to minimise overfitting proposed by Riley et al [4]; our calculation assumed the model would have a modest Nagelkerke R^2 of 25%, so with an outcome proportion of 0.435, our 12 variables corresponded to 15% overfitting [4]. This was a suitable compromise between increasing the number of PF variables and minimising overfitting.

References

1. Steyerberg EW, Eijkenmans MJ, Harrell FE, Habbema JDF. Prognostic Modelling with Logistic Regression Analysis: In search of a Sensible Strategy in Small Data Sets. Med Decis Making. 2001;21(1):45-56.

2. Hawkins DM. The problem of overfitting. J Chem Inf Comput Sci. 2004;44(1):1-12.

3. Peduzzi P, Concato J, Kemper E, Holfors TR, Feinstein AR. A Simulation Study of the Number of Events per Variable in Logistic Regression Analysis. J Clin Epidemiol. 1996;49(12):1373-9.

4. Riley RD, Snell KI, Ensor J, Burke DL, Harrell FE, Jr., Moons KG, et al. Minimum sample size for developing a multivariable prediction model: PART II - binary and time-to-event outcomes. Stat Med. 2018 Oct 24;38(7):1276-96.
