# Supplementary material for: The Value of Preseason Screening for Injury Prediction: The Development and Internal Validation of a Multivariable Prognostic Model to Predict Indirect Muscle Injury Risk in Elite Football (Soccer) Players
Source: Sports Med Open. 2020 May 27;6:22. doi: 10.1186/s40798-020-00249-8 (PMC7253524; doi:10.1186/s40798-020-00249-8)
Supplement: Supplementary file 2 — Additional file 2. Candidate prognostic factors that were excluded from the analysis, with reasons for exclusion. [file 40798_2020_249_MOESM2_ESM.pdf]

Additional file 2

The value of pre-season screening for injury prediction: The development and internal validation of a multivariable prognostic model to predict indirect muscle injury risk in elite football (soccer) players. Sports Medicine - Open.

Hughes, T., Riley, R.D. Sergeant, J.C., Callaghan, M.J. (2020)

Corresponding author: Tom Hughes

Email: tom.hughes.physio@manutd.co.uk

Correspondence address: Manchester United Football Club, AON Training Complex, Birch Road, Off Isherwood Road, Carrington, Manchester. UK. M31 4BH.
Tel: 0161 868 8754

Table A Candidate prognostic factors that were excluded from the analysis, with reasons for exclusion.

Type of prognostic factor	Candidate Prognostic Factor	Composite variable created	Measurement unit	Measurement method	Data type	Reason for elimination
Anthropometric	Body fat	No	Percentage	Skin callipers	Continuous	Missing data > 15%
Musculoskeletal test	Quadriceps muscle length difference*	Yes	Degrees	Ely's test using digital inclinometer	Continuous	Intra-rater ICC = 0.69(1) Inter-rater ICC = 0.66. (1)
	Mean quadriceps muscle length**	Yes	Degrees	Ely's test using goniometer	Continuous	Intra-rater ICC = 0.69(1) Inter-rater ICC = 0.66. (1)
	Toe touch in standing	No	Centimetres	Fingertips to floor distance	Continuous	Missing data > 15%
	Sacroiliac joint kinematic function	No	Subjective score	Gillets test	Categorical	Missing data > 15%
Functional movement/balance tests	Y Balance Test – anterior translation difference*	Yes	Centimetres	Y Balance Test	Continuous	Missing data >15%
	Y Balance Test – Mean anterior translation**	Yes	Centimetres	Y Balance Test	Continuous	Missing data >15%
	Y Balance Test – posteromedial translation difference*	Yes	Centimetres	Y Balance Test	Continuous	Missing data >15%
	Y Balance Test – Mean posteromedial translation**	Yes	Centimetres	Y Balance Test	Continuous	Missing data >15%
	Y Balance Test –posterolateral translation difference*	Yes	Centimetres	Y Balance Test	Continuous	Missing data >15%
	Y Balance Test – Mean posterolateral translation**	Yes	Centimetres	Y Balance Test	Continuous	Missing data >15%
	R relative tibial angles	No	Degrees	SLS measurement with Dorsavi Viperform IMU	Continuous	Within-session ICCs=0.27-0.75<br><br>Between-session ICCs = 0.55-0.77(2)
	L relative tibial angles (left leg)	No	Degrees	SLS measurement with Dorsavi Viperform IMU	Continuous	Within-session ICCs= 0.27-0.75<br><br>Between-session ICCs = 0.55-0.77(2)
Strength/power tests	Upper body peak power difference*	Yes	Normalised watts per kilo ($W/kg^{-0.67}$)	Double horizontal press using a Keiser Chest Press Air 350 machine	Continuous	Missing data > 15%
	Mean upper body peak power**	Yes	Normalised watts per kilo ($W/kg^{-0.67}$)	Double horizontal press using a Keiser Chest Press Air 350 machine	Continuous	Missing data > 15%

*Key: PHE=periodic health examination; WBL=weight bearing lunge; CMJ=countermovement jump; PROM=passive range of movement; ICC=intraclass correlation coefficient; SLR= straight leg raise; SLS = single leg squat; BMI= body mass index; f=force; W= watts; (note that $W/kg^{-0.67}$ has a scaling factor to normalise power to body mass) Kg=kilos; IMU= inertial measurement units; m = mass; R = right; L = left; Note: composite factors are identified in the table with * denotes between limb differences and **denotes combined mean values of both limbs*

Table B Candidate prognostic factors that were eligible for inclusion but not selected for use in model development.

Note – these remaining factors will be utilised in a forthcoming exploratory study which aims to establish their association with indirect muscle injuries in elite football (soccer) players.

Type of prognostic factor	Candidate Prognostic factor	Composite prognostic factor	Measurement unit	Measurement method	Data type	Reliability (if applicable/available)
Anthropometric	Height	No	Centimetres	Standing height measure	Continuous	-
	Weight	No	Kilograms	Digital scales	Continuous	-
Medical history	Frequency of previous foot or ankle injuries within 3 years prior to PHE.	No	Count	Medical records	Continuous	-
	Most recent previous foot or ankle injury within 3 years prior to PHE.	No	Never,<br>< 6 months,<br>6-12 months,<br>> 12 months	Medical records	Categorical	-
	Frequency of previous hip or groin injuries within 3 years prior to PHE.	No	Count	Medical records	Continuous	-
	Most recent previous hip or groin injury within 3 years prior to PHE.	No	Never,<br>< 6 months,<br>6-12 months,<br>> 12 months	Medical records	Categorical	-
	Frequency of previous knee injuries within 3 years prior to PHE.	No	Count	Medical records	Continuous	-
	Most recent previous knee injury within 3 years prior to PHE.	No	Never,<br>< 6 months,<br>6-12 months,<br>> 12 months	Medical records	Categorical	-
	Frequency of previous shoulder injuries within 3 years prior to PHE.	No	Count	Medical records	Continuous	-
	Most recent previous shoulder injury within 3 years prior to PHE.	No	Never,<br>< 6 months,<br>6-12 months,<br>> 12 months	Medical records	Categorical	-
	Frequency of previous lumbar spine injuries within 3 years prior to PHE.	No	Count	Medical records	Continuous	-
	Most recent previous lumbar spine injury within 3 years prior to PHE.	No	Never,<br>< 6 months,<br>6-12 months,<br>> 12 months	Medical records	Categorical	-
	Frequency of previous iliopsoas IMIs within 3 years prior to PHE.	No	Count	Medical records	Continuous	-
	Most recent previous iliopsoas IMIs within 3 years prior to baseline PHE.	No	Never,<br>< 6 months,<br>6-12 months,<br>> 12 months	Medical records	Categorical	-
	Frequency of previous adductor IMIs within 3 years prior to PHE.	No	Count	Medical records	Continuous	-
	Most recent previous adductor IMIs within 3 years prior to PHE.	No	Never,<br>< 6 months,<br>6-12 months,<br>> 12 months	Medical records	Categorical	-
	Frequency of previous hamstring IMIs within 3 years prior to PHE.	No	Count	Medical records	Continuous	-
	Most recent previous hamstring IMIs within 3 years prior to PHE.	No	Never,<br>< 6 months,<br>6-12 months,<br>> 12 months	Medical records	Categorical	-
	Frequency of previous quadriceps IMIs within 3 years prior to PHE.	No	Count	Medical records	Continuous	-
	Most recent previous quadriceps IMIs within 3 years prior to PHE.	No	Never,<br>< 6 months,<br>6-12 months,<br>> 12 months	Medical records	Categorical	-
	Frequency of previous calf IMIs within 3 years prior to PHE.	No	Count	Medical records	Continuous	-
	Most recent previous calf IMIs within 3 years prior to PHE.	No	Never,<br>< 6 months,<br>6-12 months,<br>> 12 months	Medical records	Categorical	-
Musculoskeletal tests	Mean PROM hip joint internal rotation**	Yes	Degrees	Digital inclinometer	Continuous	Intra-rater ICC = 0.90 (3)
	Mean PROM hip joint external rotation**	Yes	Degrees	Digital inclinometer	Continuous	Intra-rater ICC = 0.90 (3)
	Mean hip flexor muscle length**	Yes	Degrees	Thomas Test for using digital inclinometer	Continuous	Inter-rater ICC = 0.89 (4)
	Mean hamstring muscle length /neural mobility**	Yes	Degrees	SLR using digital inclinometer	Continuous	Intra-rater ICC = 0.95-0.98 (5)<br>Inter-rater ICC = 0.80-0.97 (5)
	Mean calf muscle length**	Yes	Degrees	WBL using digital inclinometer	Continuous	Inter-rater ICC = 0.80-0.95 (6, 7)<br>Intra-rater ICC = 0.88 (7)
Strength/power test	Maximal loaded leg extension power difference*	Yes	Normalised watts per kilo ($W/kg^{-0.67}$)	Double leg press test using a Keiser Air 300 machine	Continuous	Test-retest ICC = 0.886 (8)
	Mean of maximal loaded leg extension power**	Yes	Normalised watts per kilo ($W/kg^{-0.67}$)	Double leg press test using a Keiser Air 300 machine	Continuous	Test-retest ICC = 0.886 (8)
	Loaded maximal leg extension velocity difference*	Yes	Peak velocity ($m.s^{-1}$)	Double leg press test using a Keiser Air 300 machine	Continuous	Test-retest ICC = 0.792 (8)
	Mean of maximal loaded leg extension velocity**	Yes	Peak velocity ($m.s^{-1}$)	Double leg press test using a Keiser Air 300 machine	Continuous	Test-retest ICC = 0.792(8)

	Loaded maximal leg extension force difference*	Yes	Normalised peak force (N/Kg$^{-0.67}$)	Double leg press test using a Keiser Air 300 machine	Continuous	Test-retest ICC = 0.914(8)
	Mean of maximal loaded leg extension force**	Yes	Normalised peak velocity (N/Kg$^{-0.67}$)	Double leg press test using a Keiser Air 300 machine	Continuous	Test-retest ICC = 0.914(8)
	CMJ force per kilogram of body mass	No	Force per kg (N/kg)	CMJ using force plates	Continuous	-
	CMJ height	No	Centimetres	CMJ using force plates	Continuous	Test-retest ICC = 0.80-0.88.(9)

*Key: PF= prognostic factor; PHE=periodic health examination; WBL=weight bearing lunge; CMJ=countermovement jump; PROM=passive range of movement; ICC=intraclass correlation coefficient; SLR= straight leg raise; BMI= body mass index ;f=force; Kg=kilos; m = mass; m.s $^{-1}$= metres/second; Note: composite factors are identified in the table with * denoting between limb differences and **denoting combined mean values of both limbs.*

References:

1. Peeler J, Anderson JE. Reliability of the Ely's test for assessing rectus femoris muscle flexibility and joint range of motion. J Orth Res, 2008;26(6):793-9.

2. Hughes T, Jones RK, Starbuck C, Picot J, Sergeant JC, Callaghan MJ. Are tibial angles measured with inertial sensors useful surrogates for frontal plane projection angles measured using 2-dimensional video analysis during single leg squat tasks? A reliability and agreement study in elite football (soccer) players. J Electromyogr Kinesiol. 2019;44:21-30.

3. Roach S, San Juan JG, Suprak DN, Lyda MA. Concurrent validity of digital inclinometer and universal goniometer assessing passive hip mobility in healthy subjects. Int J Sports Phys Therapy. 2013;8(5):680-8.

4. Clapis PA, Davis SM, Davis RO. Reliability of inclinometer and goniometric measurements of hip extension flexibility using the modified Thomas test. Phys Theory Pract. 2008;24(2):135-41.

5. Gabbe BJ, Bennell KL, Wajswelner H, Finch CF. Reliability of common lower extremity musculoskeletal screening tests. Phys Ther Sport. 2004;5(2):90-7.

6. Williams CM, Caserta AJ, Haines TP. The TiltMeter app is a novel and accurate measurement tool for the weight bearing lunge test. J Sci Med Sport. 2013;16(5):392-5.

7. Munteanu SE, Strawhorn AB, Landorf KB, Bird AR, Murley GS. A weightbearing technique for the measurement of ankle joint dorsiflexion with the knee extended is reliable. J Sci Med Sport. 2009;12(1):54-9.

8. Redden J, Stokes K, Williams S. Establishing the Reliability and Limits of Meaningful Change of Lower Limb Strength and Power Measures during Seated Leg Press in Elite Soccer Players. J Sports Sci Med. 2018;17:539-46.

9. Slinde F, Suber C, Suber L, Edwen CE, Svantesson U. Test–retest reliability of three different countermovement jumping tests. J Strength Cond Res. 2008;22(2):640-3.
