# Supplementary material for: The Value of Preseason Screening for Injury Prediction: The Development and Internal Validation of a Multivariable Prognostic Model to Predict Indirect Muscle Injury Risk in Elite Football (Soccer) Players
Source: Sports Med Open. 2020 May 27;6:22. doi: 10.1186/s40798-020-00249-8 (PMC7253524; doi:10.1186/s40798-020-00249-8)
Supplement: Supplementary file 3 — Additional file 3. Anthropometric parameters and all included candidate PFs characteristics for participants included in the sensitivity analysis. [file 40798_2020_249_MOESM3_ESM.pdf]

Additional file 3

The value of pre-season screening for injury prediction: The development and internal validation of a multivariable prognostic model to predict indirect muscle injury risk in elite football (soccer) players. Sports Medicine - Open.

Hughes, T., Riley, R.D. Sergeant, J.C., Callaghan, M.J. (2020)

Corresponding author: Tom Hughes

Email: tom.hughes.physio@manutd.co.uk

Correspondence address: Manchester United Football Club, AON Training Complex, Birch Road, Off Isherwood Road, Carrington, Manchester. UK. M31 4BH.
Tel: 0161 868 8754

Table A Anthropometric parameters and all included candidate PFs characteristics for participants included in the sensitivity analysis

Characteristic/candidate prognostic factors	*Measurement method*	*Data type*	*Freq. (%) if categorical*	*Min*	*Lower quartile*	*Median*	*Upper quartile*	*Max*	*Missing values n (%)*
**Anthropometrics**									
Age at PHE (years)	Birthdate	Cont.	-	16.01	17.64	19.20	24.29	39.59	0 (0)
Height (cm)	Standing height	Cont.	-	164.3	176.1	180.0	185.5	195.0	15 (5.77)
Weight (kg)	Digital scales	Cont.	-	56.8	68.9	73.1	80.0	94.00	16 (6.15)
BMI (Kg/m^2)	Calculated using the formula: Kg/m^2	Cont.	-	18.1	21.7	22.6	23.4	29.1	20 (7.69)
**Past medical history**									
Freq. of previous IMIs in 3 years prior to PHE	Medical records	Dis./cont.	-	0	0	1.0	2.0	7.0	0 (0)
Most recent previous IMI in 3 years prior to PHE									
Never	Medical records	Cat.	119 (45.77)	-	-	-	-	-	0 (0)
<6 months	Medical records	Cat.	41 (15.77)	-	-	-	-	-	0 (0)
6-12 months	Medical records	Cat.	43 (16.54)	-	-	-	-	-	0 (0)
>12 months	Medical records	Cat.	57 (21.92)	-	-	-	-	-	0 (0)
**Musculoskeletal Examination**									
PROM hip internal rotation difference (deg.)	Supine ROM with digital inclinometer	Cont.	-	-25.0	-3.0	0.0	5.0	22.0	15 (5.77)
PROM hip external rotation difference (deg.)	Supine ROM with digital inclinometer	Cont.	-	-20.0	-5.0	0.0	4.4	22.0	15 (5.77)
Hip flexor length difference (deg.)	Thomas test with digital inclinometer	Cont.	-	-20.0	-2.0	0.0	2.9	14.00	18 (6.92)
Hamstring length /neural mobility difference (deg.)	SLR with digital inclinometer	Cont.	-	-20.0	0.0	0.0	0.0	15.00	15 (5.77)
Calf muscle length difference (deg.)	WBL with digital inclinometer	Cont.	-	-20.0	-2.0	0.0	3.0	15.0	15 (5.77)
**Lower Extremity Power**									
CMJ power (Watts)	CMJ using force platform	Cont.	-	2625.0	3670.0	4121.0	4619.0	6327.0	35 (13.46)

Key: PHE=periodic health examination; I-IMI=index indirect muscle injury; IMI= indirect muscle injury; IQR= interquartile range; n = observations; Freq= frequency; WBL=weight bearing lunge; CMJ=countermovement jump; PROM=passive range of movement; deg. = degrees; SLR= straight leg raise; BMI= body mass index; kg/m^2 = kilograms/body height (metres) squared; *cm = centimetres; Kg=kilograms; Cont.=continuous; dis./cont.= discrete treated as continuous; cat.= categorical. Note that for the Musculoskeletal Examination factors, positive values indicate greater left limb values compared to right limb values; negative values indicate greater right limb values compared to left limb values.*

Table B Frequency of included participant-seasons and I-IMI outcomes after exclusion of players transferred on loan or permanently, per season (sensitivity analysis).

			Season			
	1 (2013/2014)	*2 (2014/2015)*	*3 (2015/2016)*	*4 (2016/2017)*	*5 (2017/2018)*	*Total*
Included participant-seasons	41	52	54	54	59	260
Participant-seasons with an I-IMI outcome (%)	23 (56.10)	19 (36.54)	27 (50.00)	28 (51.85)	32 (54.24)	129 (49.60)
