# Supplementary material for: The Value of Preseason Screening for Injury Prediction: The Development and Internal Validation of a Multivariable Prognostic Model to Predict Indirect Muscle Injury Risk in Elite Football (Soccer) Players
Source: Sports Med Open. 2020 May 27;6:22. doi: 10.1186/s40798-020-00249-8 (PMC7253524; doi:10.1186/s40798-020-00249-8)
Supplement: Supplementary file 5 — Additional file 5. Results of the full multivariable logistic regression model and the model after variable selection – Primary complete case analysis. [file 40798_2020_249_MOESM5_ESM.pdf]

Additional file 5

The value of pre-season screening for injury prediction: The development and internal validation of a multivariable prognostic model to predict indirect muscle injury risk in elite football (soccer) players. Sports Medicine - Open.

Hughes, T., Riley, R.D. Sergeant, J.C., Callaghan, M.J. (2020)

Corresponding author: Tom Hughes

Email: tom.hughes.physio@manutd.co.uk

Correspondence address: Manchester United Football Club, AON Training Complex, Birch Road, Off Isherwood Road, Carrington, Manchester. UK. M31 4BH.
Tel: 0161 868 8754

Results of the full multivariable logistic regression model and the model after variable selection – Primary complete case analysis

	Full model							***Parsimonious model (after variable selection)***						
Candidate prognostic factors	**β†**	***95% CI***	***SE***	***OR***	***95% CI***	***SE***	***P Value***	**β†**	***95% CI***	***SE***	***OR***	***95% CI***	***SE***	***P Value***
**Anthropometrics**														
Age at PHE (years)	**0.094**	**0.022 to 0.166**	**0.037**	**1.098**	**1.022 to 1.180**	**0.040**	**0.010**	**0.087**	**0.023 to 0.151**	**0.033**	**1.091**	**1.023 to 1.163**	**0.036**	**0.008**
BMI (Kg/m^2)	-0.087	-0.271 to 0.098	0.094	0.917	0.762 to 1.103	0.086	0.357	-	-	-	-	-	-	-
**Past medical history**														
Freq. of previous IMIs in 3 years prior to PHE	**0.403**	**0.060 to 0.746**	**0.175**	**1.497**	**1.062 to 2.109**	**0.262**	**0.021**	**0.255**	**0.043 to 0.468**	**0.108**	**1.291**	**1.044 to 1.597**	**0.140**	**0.018**
Most recent previous IMI in 3 years prior to PHE														
Never	ref	ref	ref	ref	ref	ref	ref	-	-	-	-	-	-	-
<6months	-0.245	-1.331 to 0.840	0.554	0.783	0.264 to 2.317	0.433	0.658	-	-	-	-	-	-	-
6-12 months	-0.548	-1.502 to 0.406	0.487	0.578	0.223 to 1.501	0.281	0.260	-	-	-	-	-	-	-
>12 months	-0.527	-1.385 to 0.332	0.438	0.590	0.250 to 1.394	0.259	0.229	-	-	-	-	-	-	-
**Musculoskeletal Examination**														
PROM hip internal rotation difference (deg.)	0.010	-0.029 to 0.048	0.020	1.010	0.972 to 1.050	0.020	0.615	-	-	-	-	-	-	-
PROM hip external rotation difference (deg.)	0.021	-0.016 to 0.059	0.019	1.022	0.984 to 1.061	0.020	0.267	-	-	-	-	-	-	-
Hip flexor length difference (deg.)	0.028	-0.036 to 0.091	0.032	1.028	0.965 to 1.095	0.033	0.393	-	-	-	-	-	-	-
Hamstring length /neural mobility difference (deg.)	-0.022	-0.107 to 0.064	0.044	0.979	0.898 to 1.066	0.043	0.620	-	-	-	-	-	-	-
Calf muscle length difference (deg.)	0.033	-0.024 to 0.091	0.029	1.034	0.976 to 1.095	0.030	0.252	-	-	-	-	-	-	-
**Lower Extremity Power**														
CMJ power (Watts)	0.000	0.000 to 0.001	0.000	1.000	1.000 to 1.001	0.000	0.481	-	-	-	-	-	-	-
Intercept	-1.100	-4.515 to 2.314	1.742	-	-	-	-	-2.337	-3.640 to -1.033	0.665	-	-	-	-
Model Performance Statistics	***Apparent Performance (95% CI)***							***Apparent performance (95%CI) – before validation***			***Optimism-adjusted performance – after validation***			
Nagelkerke R^2	0.135							0.102			0.069			
Calibration slope	1.000 (0.580 to 1.420)							1.000 (0.535 to 1.464)			0.678 (0.213 to 1.142)			
CITL	0.000 (-0.256 to 0.256)							0.000 (-0.253 to 0.253)			-0.005 (-0.248 to 0.248)			
C-index	0.675 (0.610 to 0.741)							0.632 (0.564 to 0.701)			0.573 (0.505 to 0.642)			

Key: *β= Beta (regression) coefficient; SE= standard error; CI=confidence interval; OR=odds ratio; PHE= periodic health examination; Freq.= frequency; IMI= indirect muscle injury; deg.=degrees; BMI= body mass index; kg/m^2 = kilograms/body height squared; ref= reference category; - = not applicable; exp= exponentiate;* ***†=*** *β values are expressed per one-unit increase for all continuous variables, and according to category for the most recent IMI within 3 years prior to PHE.* ***Note:*** *Factors in* **bold** *indicate significance at the 0.157 level (equivalent to Akaike's information criterion).*
