# Supplementary material for: The Value of Preseason Screening for Injury Prediction: The Development and Internal Validation of a Multivariable Prognostic Model to Predict Indirect Muscle Injury Risk in Elite Football (Soccer) Players
Source: Sports Med Open. 2020 May 27;6:22. doi: 10.1186/s40798-020-00249-8 (PMC7253524; doi:10.1186/s40798-020-00249-8)
Supplement: Supplementary file 6 — Additional file 6. Results of the full multivariable logistic regression model and the model after variable selection – Sensitivity analysis using imputed data. [file 40798_2020_249_MOESM6_ESM.pdf]

Additional file 6

The value of pre-season screening for injury prediction: The development and internal validation of a multivariable prognostic model to predict indirect muscle injury risk in elite football (soccer) players. Sports Medicine - Open.

Hughes, T., Riley, R.D. Sergeant, J.C., Callaghan, M.J. (2020)

Corresponding author: Tom Hughes

Email: tom.hughes.physio@manutd.co.uk

Correspondence address: Manchester United Football Club, AON Training Complex, Birch Road, Off Isherwood Road, Carrington, Manchester. UK. M31 4BH.
Tel: 0161 868 8754

Results of the full multivariable logistic regression model and the model after variable selection – Sensitivity analysis using imputed data

	Full model							*Parsimonious model (after backwards selection)*						
Candidate prognostic factors	**β†**	***95% CI***	***SE***	***OR***	***95% CI***	***SE***	***P Value***	**β†**	***95% CI***	***SE***	***OR***	***95% CI***	***SE***	***P Value***
**Anthropometrics**														
Age at PHE (years)	**0.105**	**0.033 to 0.177**	**0.037**	**1.110**	**1.033 to 1.194**	**0.041**	**0.004**	**0.113**	**0.049 to 0.177**	**0.033**	**1.119**	**1.050 to 1.193**	**0.037**	**0.001**
BMI (Kg/m^2)	0.030	-0.168 to 0.227	0.101	1.030	0.845 to 1.255	0.104	0.770	-	-	-	-	-	-	
**Past medical history**														
Freq. of previous IMIs in 3 years prior to PHE	**0.340**	**-0.03 to 0.712**	**0.190**	**1.404**	**0.968 to 2.038**	**0.267**	**0.074**	**0.263**	**0.033 to 0.493**	**0.118**	**1.301**	**1.033 to 1.638**	**0.153**	**0.025**
Most recent previous IMI in 3 years prior to PHE														
Never	ref	ref	ref	ref	ref	ref	Ref	-	-	-	-	-	-	-
<6 months	0.014	-1.079 to 1.108	0.558	1.014	0.340 to 3.029	0.566	0.979	-	-	-	-	-	-	-
6-12 months	-0.521	-1.482 to 0.441	0.491	0.594	0.227 to 1.553	0.291	0.288	-	-	-	-	-	-	-
>12 months	-0.140	-1.057 to 0.777	0.468	0.869	0.347 to 2.175	0.407	0.764	-	-	-	-	-	-	-
**Musculoskeletal Examination**														
PROM hip internal rotation difference (deg.)	0.001	-0.041 to 0.044	0.022	1.001	0.960 to 1.045	0.022	0.952	-	-	-	-	-	-	-
PROM hip external rotation difference (deg.)	0.013	-0.028 to 0.054	0.021	1.013	0.973 to 1.055	0.021	0.532	-	-	-	-	-	-	-
Hip flexor length difference (deg.)	0.043	-0.023 to 0.108	0.033	1.044	0.977 to 1.115	0.035	0.201	-	-	-	-	-	-	-
Hamstring length /neural mobility difference (deg.)	-0.001	-0.084 to 0.083	0.043	0.999	0.919 to 1.087	0.043	0.990	-	-	-	-	-	-	-
Calf muscle length difference (deg.)	0.009	-0.049 to 0.066	0.029	1.009	0.953 to 1.068	0.029	0.768	-	-	-	-	-	-	-
**Lower Extremity Power**														
CMJ power (Watts)	0.000	-0.001 to 0.000	0.000	1.000	0.999 to 1.000	0.000	0.825	-	-	-	-	-	-	-
Intercept	-2.877	-6.312 to 0.0558	1.752	-	-	-	-	-2.647	-3.948 to -1.347	0.663	-	-	-	-
Model Performance Statistics	***Apparent performance with 95% CI (if applicable)***							***Apparent performance with 95% CI (if applicable)- before validation***			***Optimism-adjusted performance with 95% CI – after validation***			
Nagelkerke R^2	0.168							0.145			0.104			
Calibration slope	1.000 (0.620 to 1.380)							1.000 (0.598 to 1.402)			0.715 (0.313to 1.117)			
CITL	0.000 (-0.259 to 0.259)							0.000 (-0.257 to 0.257)			-0.009 (-0.248 to 0.248)			
C-index	0.705 (0.642 to 0.768)							0.691 (0.626 to 0.754)			0.639 (0.574 to 0.70.5)			

Key: *β= Beta (regression) coefficient; SE= standard error; CI=confidence interval; OR=odds ratio; PHE= periodic health examination; Freq.= frequency; IMI= indirect muscle injury; deg.=degrees; BMI= body mass index; kg/m^2 = kilograms/body height squared; ref= reference category;* ***-*** *= not applicable; exp= exponentiate;* ***†=*** *β values are expressed per one-unit increase for all continuous variables, and according to category for the most recent IMI within 3 years prior to PHE.* ***Note:*** *Factors in* ***bold*** *indicate significance at the 0.157 level (equivalent to Akaike's information criterion).*
