# Supplementary material for: The Value of Preseason Screening for Injury Prediction: The Development and Internal Validation of a Multivariable Prognostic Model to Predict Indirect Muscle Injury Risk in Elite Football (Soccer) Players
Source: Sports Med Open. 2020 May 27;6:22. doi: 10.1186/s40798-020-00249-8 (PMC7253524; doi:10.1186/s40798-020-00249-8)
Supplement: Supplementary file 7 — Additional file 7. Results of the full multivariable logistic regression model and the model after variable selection – Sensitivity analysis using complete case data. [file 40798_2020_249_MOESM7_ESM.pdf]

Additional File 7

The value of pre-season screening for injury prediction: The development and internal validation of a multivariable prognostic model to predict indirect muscle injury risk in elite football (soccer) players. Sports Medicine - Open.

Hughes, T., Riley, R.D. Sergeant, J.C., Callaghan, M.J. (2020)

Corresponding author: Tom Hughes

Email: tom.hughes.physio@manutd.co.uk

Correspondence address: Manchester United Football Club, AON Training Complex, Birch Road, Off Isherwood Road, Carrington, Manchester. UK. M31 4BH.
Tel: 0161 868 8754

Results of the full multivariable logistic regression model and the model after variable selection – Sensitivity analysis using complete case data

	Full model							***Parsimonious model (after variable selection)***						
Candidate prognostic factors	**β†**	***95% CI***	***SE***	***OR***	***95% CI***	***SE***	***P Value***	**β†**	***95% CI***	***SE***	***OR***	***95% CI***	***SE***	***P Value***
**Anthropometrics**														
Age at PHE (years)	**0.120**	**0.037 to 0.204**	**0.043**	**1.128**	**1.037 to 1.227**	**0.048**	**0.005**	**0.120**	**0.045 to 0.195**	**0.038**	**1.127**	**1.046 to 1.215**	**0.043**	**0.002**
BMI (Kg/m^2)	-0.005	-0.211 to 0.201	0.105	0.995	0.810 to 1.222	0.104	0.961	-	-	-	-	-	-	-
**Past medical history**														
Freq. of previous IMIs in 3 years prior to PHE	**0.449**	**0.005 to 0.892**	**0.226**	**1.566**	**1.005 to 2.441**	**0.355**	**0.048**	**0.307**	**0.045 to 0.569**	**0.134**	**1.359**	**1.046 to 1.766**	**0.046**	**0.022**
Most recent previous IMI in 3 years prior to PHE														
Never	ref	ref	ref	ref	ref	ref	ref	-	-	-	-	-	-	-
<6 months	-0.187	-1.406 to 1.033	0.622	0.830	0.245 to 2.808	0.516	0.764	-	-	-	-	-	-	-
6-12 months	-0.642	-1.710 to 0.425	0.545	0.526	0.181 to 1.530	0.287	0.238	-	-	-	-	-	-	-
>12 months	-0.243	-1.269 to 0.783	0.524	0.784	0.281 to 2.188	0.411	0.642	-	-	-	-	-	-	-
**Musculoskeletal Examination**								-	-	-	-	-	-	-
PROM hip internal rotation difference (deg.)	0.120	-0.035 to 0.053	0.022	1.009	0.965 to 1.054	0.023	0.702	-	-	-	-	-	-	-
PROM hip external rotation difference (deg.)	0.011	-0.032 to 0.053	0.022	1.011	0.969 to 1.055	0.022	0.622	-	-	-	-	-	-	-
Hip flexor length difference (deg.)	0.036	-0.037 to 0.110	0.038	1.037	0.963 to 1.116	0.039	0.335	-	-	-	-	-	-	-
Hamstring length /neural mobility difference (deg.)	-0.013	-0.109 to 0.083	0.049	0.987	0.897 to 1.086	0.048	0.787	-	-	-	-	-	-	-
Calf muscle length difference (deg.)	0.032	-0.033 to 0.096	0.033	1.032	0.968 to 1.101	0.034	0.335	-	-	-	-	-	-	-
**Lower Extremity Power**														
CMJ power (Watts)	0.000	-0.001 to 0.000	0.000	1.000	0.999 to 1.000	0.000	0.806	-	-	-	-	-	-	-
Intercept	-2.392	-6.081 to 1.297	1.882	-	-	-	-	-2.797	-4.286 to -1.309	0.759	-	-	-	-

Model Performance Statistics	***Apparent performance with 95% CI (if applicable)***	***Apparent performance with 95% CI (if applicable)- before validation***	***Optimism-adjusted performance with 95% CI- after validation***
Nagelkerke R^2	0.178	0.154	0.110
Calibration slope	1.000 (0.577 to 1.422)	1.000 (0.562 to 1.440)	0.712 (0.274 to 1.152)
CITL	0.000 (-0.285 to 0.285)	0.000 (-0.283 to 0.283)	-0.008 (-0.275 to 0.275)
C-index	0.703 (0.633 to 0.772)	0.683 (0.613 to 0.754)	0.628 (0.558 to 0.699)

Key: *β= Beta (regression) coefficient; SE= standard error; CI=confidence interval; OR=odds ratio; PHE= periodic health examination; Freq.= frequency; IMI= indirect muscle injury; deg.=degrees; BMI= body mass index; kg/m^2 = kilograms/body height squared; ref= reference category;* **-** *= not applicable; exp= exponentiate;***†=** *β values are expressed per one-unit increase for all continuous variables, and according to category for the most recent IMI within 3 years prior to PHE.* ***Note:*** *Factors in* ***bold*** *indicate significance at the 0.157 level (equivalent to Akaike's information criterion)*
