# Supplementary material for: The Value of Preseason Screening for Injury Prediction: The Development and Internal Validation of a Multivariable Prognostic Model to Predict Indirect Muscle Injury Risk in Elite Football (Soccer) Players
Source: Sports Med Open. 2020 May 27;6:22. doi: 10.1186/s40798-020-00249-8 (PMC7253524; doi:10.1186/s40798-020-00249-8)

Additional file 8

The value of pre-season screening for injury prediction: The development and internal validation of a multivariable prognostic model to predict indirect muscle injury risk in elite football (soccer) players. Sports Medicine - Open.

Hughes, T., Riley, R.D. Sergeant, J.C., Callaghan, M.J. (2020)

Corresponding author: Tom Hughes

Email: tom.hughes.physio@manutd.co.uk

Correspondence address: Manchester United Football Club, AON Training Complex, Birch Road, Off Isherwood Road, Carrington, Manchester. UK. M31 4BH.
Tel: 0161 868 8754

Apparent calibration plots for primary complete case analysis and sensitivity analyses.

Fig. A: Apparent calibration plot for parsimonious model after variable selection – primary analysis (complete case data)

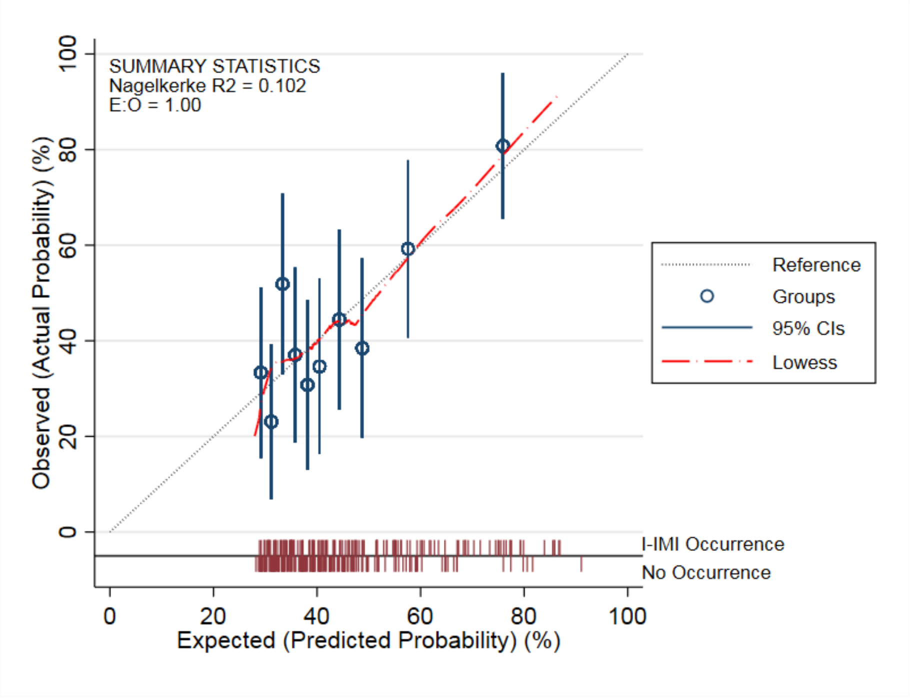

Fig. B: Apparent calibration plot for parsimonious model after variable selection – sensitivity analysis (imputed data)

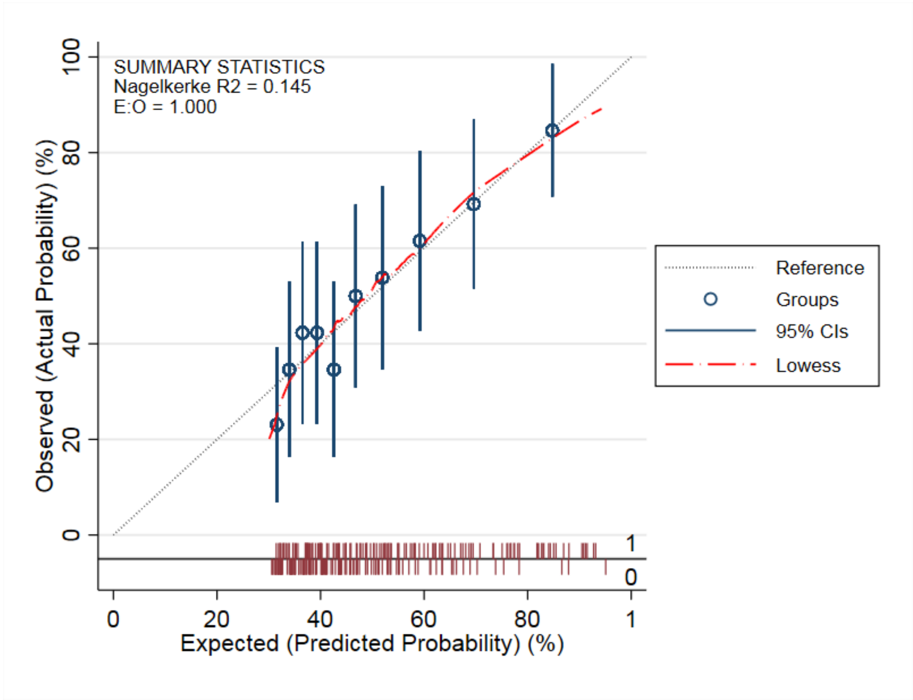

Fig. C: Apparent calibration plot for parsimonious model after variable selection – sensitivity analysis (complete case data)

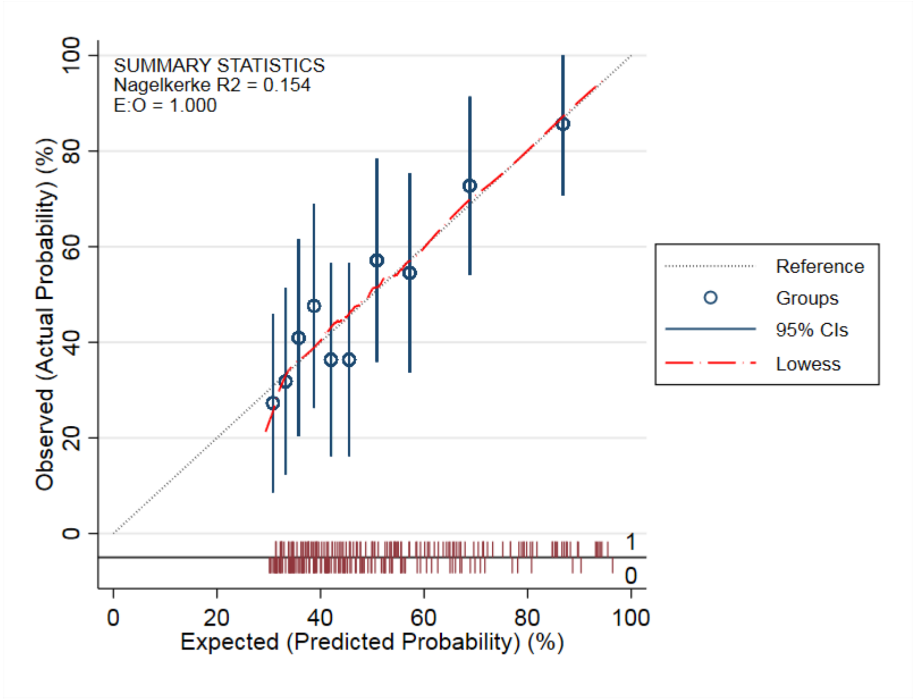

Supplement: Supplementary file 8 — Additional file 8. Apparent calibration plots for primary complete case analysis and sensitivity analyses. [file 40798_2020_249_MOESM8_ESM.pdf]
