# Supplementary material for: The Value of Preseason Screening for Injury Prediction: The Development and Internal Validation of a Multivariable Prognostic Model to Predict Indirect Muscle Injury Risk in Elite Football (Soccer) Players
Source: Sports Med Open. 2020 May 27;6:22. doi: 10.1186/s40798-020-00249-8 (PMC7253524; doi:10.1186/s40798-020-00249-8)
Supplement: Supplementary file 9 — Additional file 9. Full internal validation results for all analyses. [file 40798_2020_249_MOESM9_ESM.pdf]

Additional file 9

The value of pre-season screening for injury prediction: The development and internal validation of a multivariable prognostic model to predict indirect muscle injury risk in elite football (soccer) players. Sports Medicine - Open.

Hughes, T., Riley, R.D. Sergeant, J.C., Callaghan, M.J. (2020)

Corresponding author: Tom Hughes

Email: tom.hughes.physio@manutd.co.uk

Correspondence address: Manchester United Football Club, AON Training Complex, Birch Road, Off Isherwood Road, Carrington, Manchester. UK. M31 4BH.
Tel: 0161 868 8754

Full internal validation results for all analyses

Table A Full internal validation results - primary analysis using imputed data

Variable	Mean	SD	Min	Max
C index (Apparent)	0.685	0.030	0.612	0.773
C Index (Test)	0.632	0.014	0.587	0.664
**C Index (Optimism)**	**0.052**	**0.029**	**-0.026**	**0.128**
CITL (Apparent)	0.000	0.000	0.000	0.000
CITL (Test)	-0.009	0.130	-0.388	0.394
**CITL (Optimism)**	**0.009**	**0.130**	**-0.394**	**0.388**
Slope (Apparent)	1.000	0.000	1.000	1.000
Test Slope (Test)	0.718	0.159	0.446	1.455
**Slope (Optimism)**	**0.282**	**0.159**	**-0.455**	**0.554**

Key: SD=standard deviation; min= minimum value; max= maximum value; CITL= calibration in the large. Note: Apparent performance is the performance of the bootstrap model in the bootstrap dataset); test performance is the performance of the bootstrap model in the original dataset.

Table B Full internal validation results - primary analysis using complete case data

Variable	Mean	SD	Min	Max
C index (Apparent)	0.694	0.035	0.613	0.809
C Index (Test)	0.635	0.017	0.546	0.664
C Index (Optimism)	0.059	0.032	-0.024	0.148
CITL (Apparent)	0.000	0.000	0.000	0.000
CITL (Test)	-0.005	0.133	-0.364	0.360
**CITL (Optimism)**	0.005	0.133	-0.360	0.364
Slope (Apparent)	1.000	0.000	1.000	1.000
Test Slope (Test)	0.678	0.147	0.349	1.194
**Slope (Optimism)**	0.322	0.147	-0.194	0.651

Key: SD=standard deviation; min= minimum value; max= maximum value; CITL= calibration in the large. Note: Apparent performance is the performance of the bootstrap model in the bootstrap dataset); test performance is the performance of the bootstrap model in the original dataset.

Table C Full internal validation results - sensitivity analysis using imputed data

Variable	Mean	SD	Min	Max
C index (Apparent)	0.725	0.034	0.567	0.806
C Index (Test)	0.673	0.017	0.587	0.701
**C Index (Optimism)**	**0.052**	**0.035**	**-0.057**	**0.131**
CITL (Apparent)	1.000	0.000	1.000	1.000
CITL (Test)	0.009	0.139	-0.300	0.322
**CITL (Optimism)**	**-0.009**	**0.139**	**-0.322**	**0.300**
Slope (Apparent)	1.000	0.000	1.000	1.000
Test Slope (Test)	0.715	0.171	0.296	1.348
**Slope (Optimism)**	**0.285**	**0.171**	**-0.348**	**0.704**

Key: SD=standard deviation; min= minimum value; max= maximum value; CITL= calibration in the large. Note: Apparent performance is the performance of the bootstrap model in the bootstrap dataset); test performance is the performance of the bootstrap model in the original dataset.

Table D Full internal validation results - sensitivity analysis using complete case data

Variable	Mean	SD	Min	Max
C index (Apparent)	0.725	0.038	0.600	0.810
C Index (Test)	0.670	0.019	0.595	0.702
**C Index (Optimism)**	0.055	0.039	-0.074	0.156
CITL (Apparent)	0.000	0.000	0.000	0.000
CITL (Test)	-0.008	0.163	-0.422	0.742
**CITL (Optimism)**	0.008	0.163	-0.742	0.422
Slope (Apparent)	1.000	0.000	1.000	1.000
Test Slope (Test)	0.712	0.182	0.359	1.480
**Slope (Optimism)**	0.288	0.182	-0.480	0.641

Key: SD=standard deviation; min= minimum value; max= maximum value; CITL= calibration in the large. Note: Apparent performance is the performance of the bootstrap model in the bootstrap dataset); test performance is the performance of the bootstrap model in the original dataset.
